# Correlation analysis on epidermal growth factor receptor (EGFR) mutations and clinicopathological characteristics in lung adenocarcinomas

**DOI:** 10.3389/fonc.2025.1519150

**Published:** 2025-03-20

**Authors:** Haitao Wang, Changhua Ji, Chengjun Zhou, Hui Li

**Affiliations:** Department of Pathology, The Second Hospital of Shandong University, Jinan, Shandong, China

**Keywords:** EGFR mutation, lung adenocarcinoma, histology, pathology, molecular pathology

## Abstract

**Purpose:**

To analysis the correlation between EGFR mutations and clinicopathological features in lung adenocarcinomas.

**Methods:**

139 lung adenocarcinoma cases from the Second Hospital of Shandong University were conducted molecular detection of EGFR mutations. Multiple clinicopathological characteristics were collected and analyzed to identify the relationship with EGFR mutations. The amplification refractory mutation system (ARMS) was performed to detect the EGFR mutations.

**Results:**

During the 139 cases, 96 lung adenocarcinoma cases had EGFR mutations. EGFR mutations were associated with smoking history (P=0.0311), tumor size (P=0.0247), tumor subtype (P=0.0003), rhabdomyoid differentiation (P=0.0237) and extracellular mucus (P=0.0013).

**Conclusions:**

Smoking history, tumor size, tumor subtype, rhabdomyoid differentiation and extracellular mucus were related to EGFR mutations in lung adenocarcinoma. These histological characteristics might be meaningful to predict EGFR mutations.

## Introduction

Lung cancer is the leading cause of cancer-related mortality worldwide (1). Adenocarcinoma is the most common histological subtype of lung cancer, accounting for approximately 40% (2). Lung adenocarcinoma is a type of non-small cell lung cancer (NSCLC) that arises from glandular cells in the lung, typically in the outer or peripheral regions of the lung (3). The histological features of lung adenocarcinoma are diverse, including glandular or acinar structures, papillary structures, solid or micropapillary growth pattern and so on. In 2011, the International Association for the Study of Lung Cancer/American Thoracic Society/European Respiratory Society (IASLC/ATS/ERS) proposed the standardized classification system of lung adenocarcinoma and other lung cancer subtypes based on the histological features (4). According to the IASLC/ATS/ERS classification, the invasive non-mucinous lung adenocarcinoma can be divided into five subtypes based on the predominant architectural pattern: lepidic adenocarcinoma (LA), acinar adenocarcinoma (AA), papillary adenocarcinoma (PA), solid adenocarcinoma (SA) and micropapillary adenocarcinoma (MA). In addition, based on a study undertaken by the International Association for the Study of Lung Cancer (IASLC) Pathology Committee, a new grading system of invasive non-mucinous lung adenocarcinoma has been recommended as below: grade 1, lepidic-predominant with no or < 20% high-grade pattern (solid, micropapillary, cribriform, or complex glandular pattern); grade 2, acinar or papillary-predominant with no or < 20% high-grade pattern; grade 3: any tumor with ≥ 20% high-grade pattern. These three grades respectively correspond to well-differentiation, moderately differentiation, and poorly differentiation (5).

Lung adenocarcinoma is a highly heterogeneous disease with various genetic and molecular alterations. These genetic alterations play vital roles in the occurrence and development of lung adenocarcinoma. One of the most frequently observed genetic mutations in lung adenocarcinoma is in the EGFR gene, which codes for a transmembrane receptor tyrosine kinase (6). EGFR mutations at the tyrosine kinase domain keep EGFR in an activation state, leading to cell proliferation and growth. EGFR is overexpressed and/or mutated in a variety of human cancers, including NSCLC (7). EGFR mutations occur in approximately 15-70% of all lung adenocarcinoma cases, with a higher prevalence in non-smokers, females and individuals of Asian origin (8–15). These mutations are mostly found in exons 18-21 of the EGFR gene and result in EGFR constitutive activation, promoting the downstream signaling pathways, such as the PI3K-AKT and MAPK pathways. During these mutations, the most common mutations are exon 19 deletions and exon 21 L858R point mutation, which account for approximately 85% of all EGFR mutations in NSCLC (16, 17). Exon 19 deletions result in the loss of amino acids 747-750, while the L858R mutation leads to a substitution of arginine for leucine at the position 858 (18). These mutations occur in the tyrosine kinase domain of EGFR, which is responsible for the activation of downstream signaling pathways (19). This activation promotes tumor cell growth, survival, and metastasis, and confers resistance to chemotherapy and radiotherapy (19).

EGFR-mutated NSCLC is typically sensitive to EGFR tyrosine kinase inhibitors (TKIs), such as gefitinib, erlotinib, osimertinib, Lazertinib and almonertinib, which are small-molecule inhibitors that target the ATP-binding site of the EGFR tyrosine kinase domain (20–23). Treatment with EGFR TKIs has been shown to improve the progression-free survival (PFS) and overall response rates (ORR) compared to standard chemotherapy in patients with advanced EGFR-mutated NSCLC (24, 25).

In this research, we analyzed the relationship between EGFR mutations and the clinicopathological features of lung adenocarcinoma to better understand the function of EGFR mutations in lung adenocarcinoma. Besides the histological subtypes of lung adenocarcinoma, we also included a variety of morphological features, such as lymphocytic infiltration, extracellular mucin, fibrosis, mucinous tumor cells, tumor necrosis, abscess, hobnail cells, rhabdoid cells, spread through air spaces (STAS), abundant mitosis, prominent nucleoli, high-grade nuclei and nuclear inclusion bodies.

In addition, we also collected and analyzed the data of lymph node metastasis, pleural invasion, vascular invasion and Ki67 proliferation index.

## Materials and methods

### Patients

**Figure 1:**
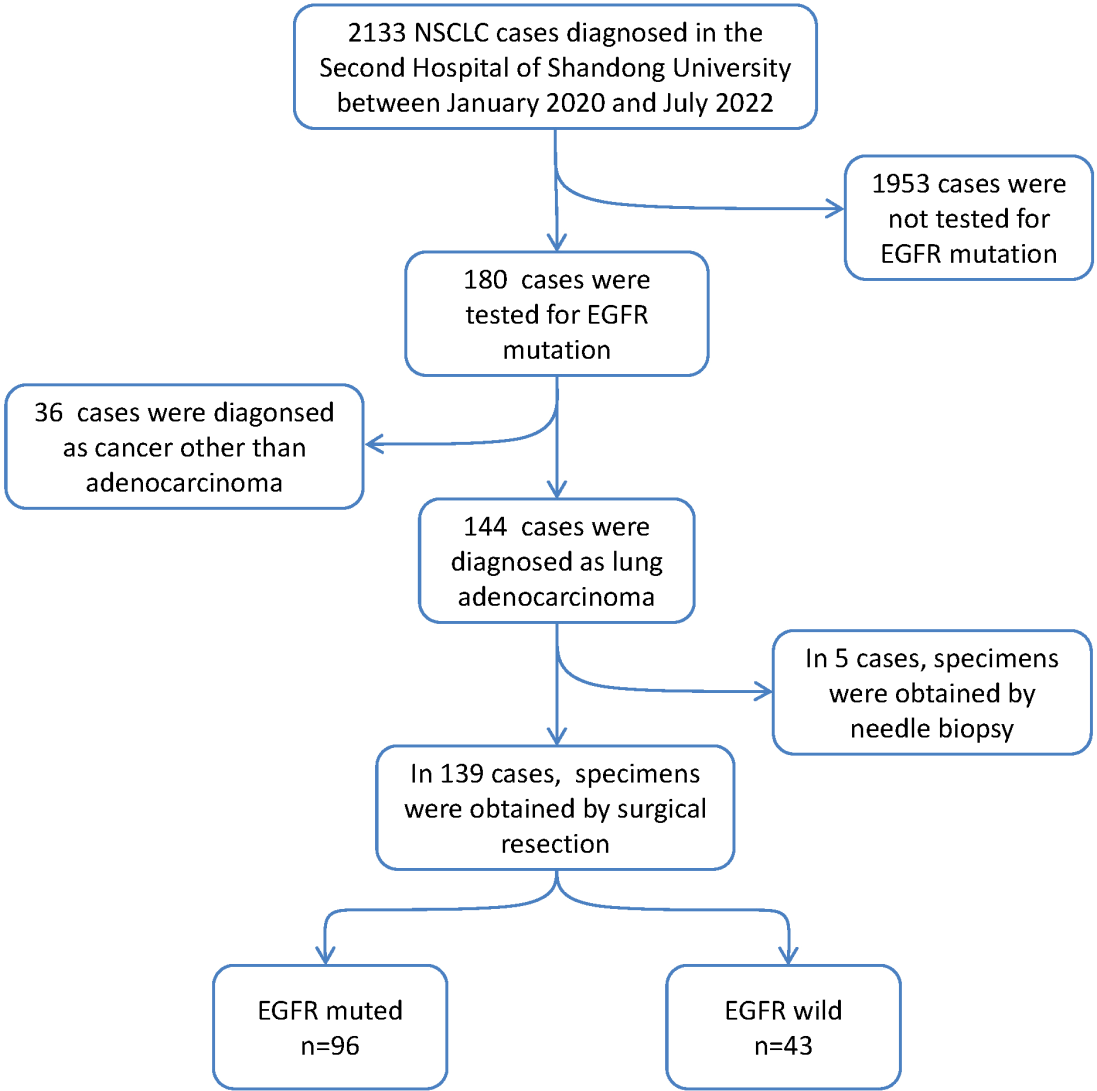
Flow charts for inclusion of patients in our study cohort.

The prospective study was performed among 139 formalin-fixed paraffin-embedded (FFPE) tumors, which were diagnosed as non-mucinous lung adenocarcinoma and tested for EGFR mutation (**Figure 1**). All specimens were obtained from resected surgery. All patients were diagnosed and treated in the Second Hospital of Shandong University between January 2020 and July 2022. None of the patients received preoperative chemoradiotherapy. The clinical information of the 139 patients is shown in **Table 1**.

**Table 1:** The correlation between EGFR mutations and the clinical features.

	N	Mutant EGFR	Wild type EGFR	P value
**Gender**				0.1690
Male	56	35	21	
Female	83	61	22	
**Age**				0.9683
>59	65	45	20	
≤59	74	51	23	
**Location**				0.0923
Left	52	32	20	
Right	86	64	22	
Left & Right	1	0	1	
**Smoking**				***0.0311***
Yes	27	14	13	
No	112	82	30	
**Hypertension**				0.3845
Yes	46	34	12	
No	93	62	31	
**Diabetes Mellitus**				0.9769
Yes	16	11	5	
No	123	85	38	
**Family History**				0.6417
Yes	12	9	3	
No	127	87	40	
**Tumor count**				0.7818
Multiple	43	29	14	
Single	96	67	29	
**Size**				***0.0247***
≤1.5cm	74	45	29	
>1.5cm	65	51	14	
**Lymphatic metastasis**				0.2384
Yes	16	9	7	
No	123	87	36	

The bold and italic values mean statistically significant.

### Histological analysis

**Figure 2:**
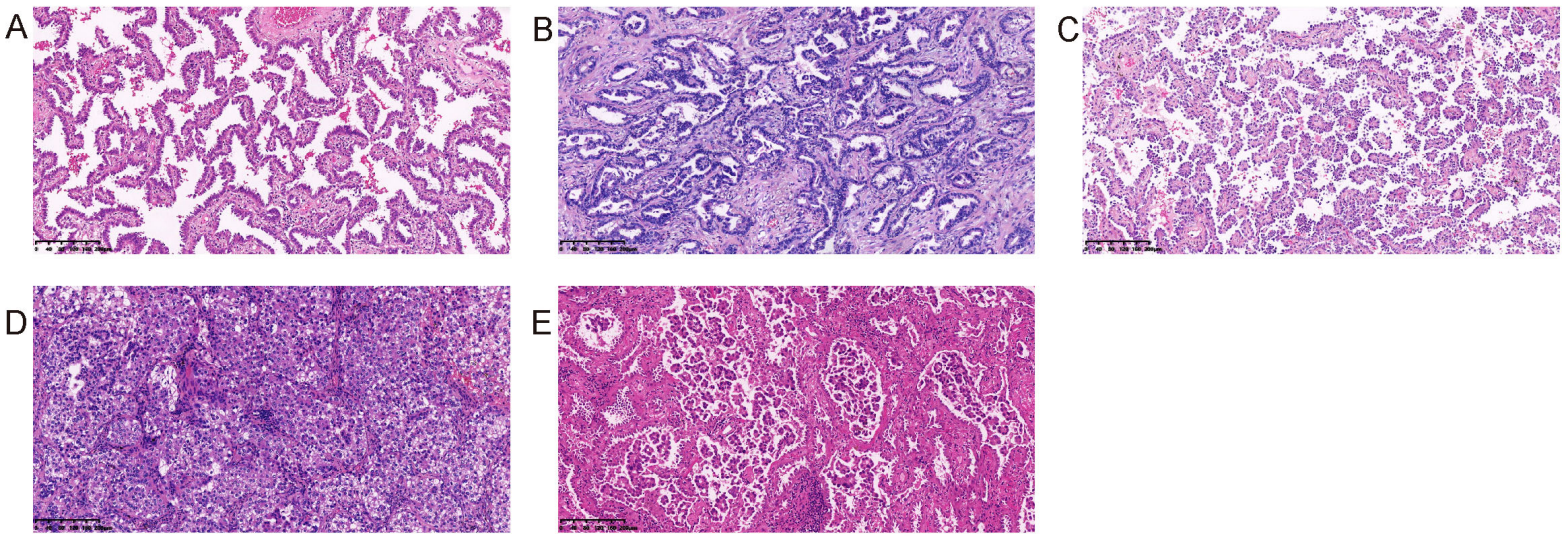
Five histological subtypes of lung adenocarcinoma. **(A)** Lepidic adenocarcinoma; **(B)** Acinar adenocarcinoma; **(C)** Papillary adenocarcinoma; **(D)** Solid adenocarcinoma; **(E)** Micropapillary adenocarcinoma.

All the pathological slides were examined and diagnosed according to the World Health Organization (WHO) classification system. The World Health Organization (WHO) classification system recognizes five subtypes of lung adenocarcinoma (**Figure 2**), which are characterized by distinct morphological features and genetic alterations (5). The most common subtype of lung adenocarcinoma is the acinar subtype, which is characterized by the presence of glandular structures that resemble acini, or grape-like clusters of cells. Other subtypes include the papillary subtype (which has finger-like projections of cells), the solid subtype (which lacks glandular structures and is composed of sheets of cells), and the lepidic subtype (which consists of predominantly bland pneumocytic cells growing along the surface of alveolar walls and subordinately an invasive component measuring > 5 mm in greatest dimension), and the micropapillary subtype (which is composed of tumor cells growing in papillary tufts forming florets that lack fibrovascular cores) (5).

**Figure 3:**
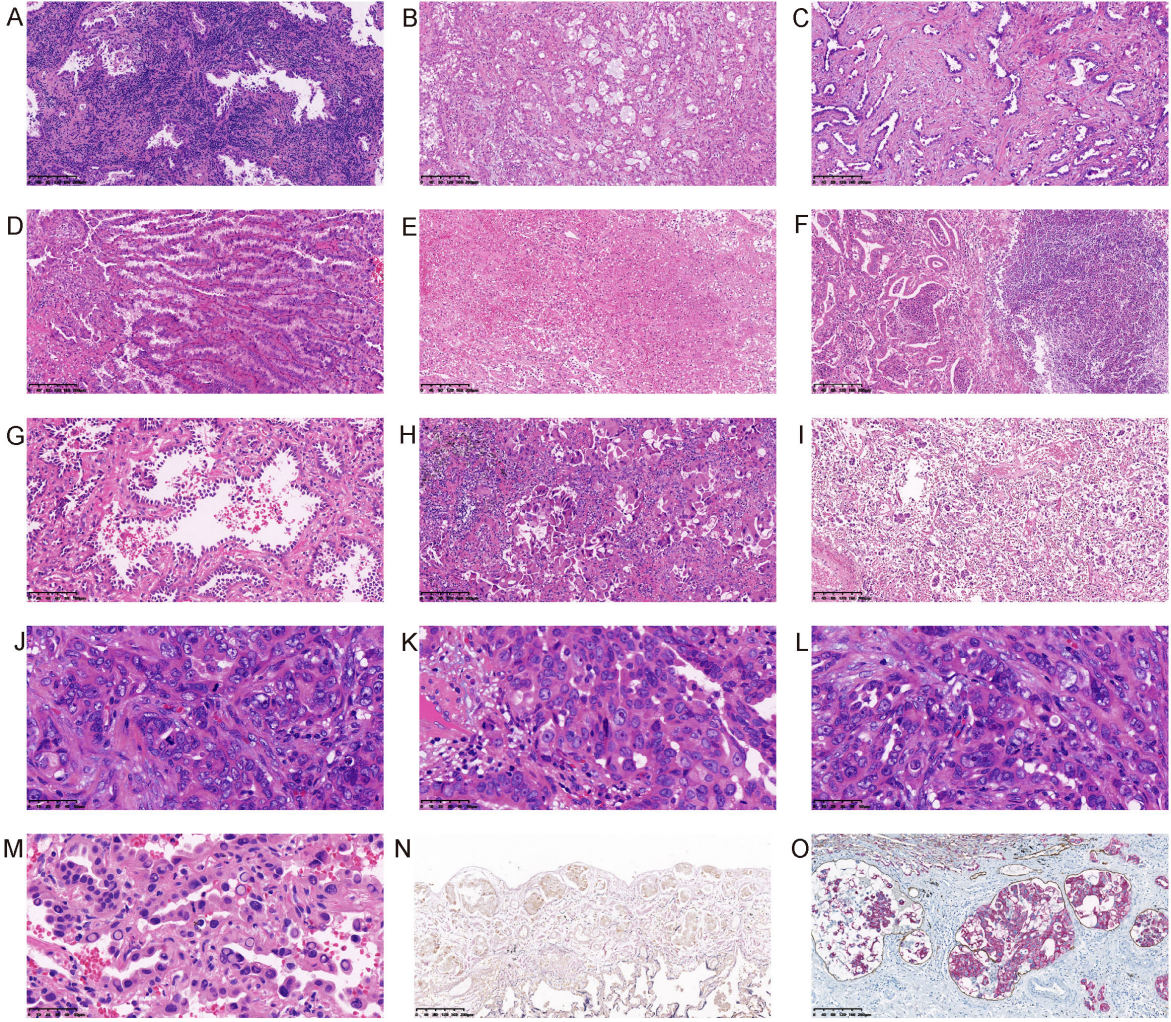
Morphological features of lung adenocarcinoma. **(A)** Lymphocytes infiltration; **(B)** Extracellular mucus; **(C)** Fibrosis; **(D)** Mucinous epithelium; **(E)** Necrosis; **(F)** Abscess; **(G)** Hobnail cell; **(H)** Rhabdomyoid differentiation; **(I)** STAS; **(J)** Mitosis; **(K)** Nucleolus; **(L)** High grade nuclear; **(M)** Intranuclear inclusion; **(N)** Pleural invasion (Elastin fiber staining showed that the elastic fibers in the visceral pleura were interrupted by tumor invasion); **(O)** Lymphatic vessel invasion (The immunohistochemical double staining of CK and D2-40 showed the intravascular cancer embolus).

The lung adenocarcinoma has many microscpical variables, such as lymphocytes infiltration, extracellular mucus, fibrosis, mucinous epithelium, necrosis, abscess, pleural invasion, vessel invasion, STAS, lymphatic metastasis, hobnail cell, rhabdomyoid differentiation, mitosis, nucleolus, high grade nuclear and nuclear inclusion (**Figure 3**). We grouped our cohort according to the presence or absence of these morphological features, and analyzed the correlation between these morphological features and the EGFR mutations. The morphological features were described as follows. Lymphocytes infiltration:diffuse or small clusters of lymphocytes infiltration. Mucinous epithelium: lung adenocarcinoma with less than 10% of mucinous components. Extracellular mucus: significant extracellular mucous. STAS: tumor cells within air spaces in the lung parenchyma beyond the edge of the main tumor (5). Rhabdomyoid differentiation: refers to tumor cells rich in eosinophilic cytoplasm with eccentric nuclei resembling striated muscle cells, which can be found in a variety of tumors, such as parathyroid carcinoma (26), breast cancer (27), ovarian cancer (28), malignant melanoma (29), renal cell carcinoma (30), and glioblastoma (31). Pleural invasion, tumor cells invading the pleural. Vessel invasion, tumor cells invading the vessels. High grade nuclear: the nucleus of the tumor cells was 5 times larger than the lymphocytes nucleus. Hobnail cell: a centrally located nucleus with a bulging appearance and a narrow base, resembling a hobnail. All the histological evaluations were performed by two senior pathologists.

### EGFR mutation analysis

Formalin-fixed paraffin-embedded (FFPE) lung cancer tissues were obtained after surgery and biopsy puncture. The DNA were extracted from the FFPE samples using the AmoyDx FFPE DNA/RNA Extraction Kit (Amoy Diagnostics, Xiamen, China). The PCR amplification was performed with the SuperARMS EGFR Mutation Detection Kit (Amoy Diagnostics, Xiamen, China) on cobas z480 analyzer (Roche). The EGFR mutations at exons 18–21 were analyzed. All kits and devices were used following the manufacturer’s protocol.

### Immunohistochemistry

The immunohistochemistry (IHC) is a routine technique that can provide additional information of marker proteins in lung adenocarcinoma, such as TTF-1 (thyroid transcription factor 1), Napsin A and CK7 (cytokeratin 7). Other indicators were also tested, such as P63, Ki67, and so on. These markers can help distinguish lung adenocarcinoma from other lung cancer subtypes and the metastatic tumors. The IHC was performed as previously described (32). The immunohistochemical double staining is a method of simultaneous detection the expression of two different antigens in the same tissue slice. This technique involves two primary antibodies, followed by two different secondary antibodies conjugated to different chromogens. In this study, the method was used to simultaneously stain CK and D2-40 in the same tissue section, so as to distinctly determine whether the tumor cells invaded the lymphatic vessels.

### Statistical analysis

The correlations between clinicopathological characteristics and EGFR mutations were analyzed using the chi-square test. All statistical analyses were performed using the Graphpad software. P<0.05 was considered as statistically significant.

## Results

### Clinical characteristics of the cohort

Clinical information of 139 patients was shown in **Table 1**. There were 56 males (40.3%) and 83 females (59.7%). The median age at diagnosis was 59 years (range 33-83 years). 27 (19.4%) patients were current or former smokers, and 112 (80.6%) were non-smokers. 46 (33.1%) patients had a hypertension history and 16(11.5%) patients had a history of diabetes. 12 (8.6%) patients had a family history of lung cancer in their immediate family, and 127 (91.4%) patients denied a family history of lung cancer. 52 (37.4%) cases were located in the left lung, 86 (61.9%) cases in the right lung, and 1 (0.7%) case in both lungs. 43 (30.9%) patients had multiple tumors. The median size of the tumors was 1.5 cm (range 0.5-8cm). 16 cases (11.5%) were with lymphatic metastasis.

### Correlation between EGFR mutations and clinical features

**Figure 4:**
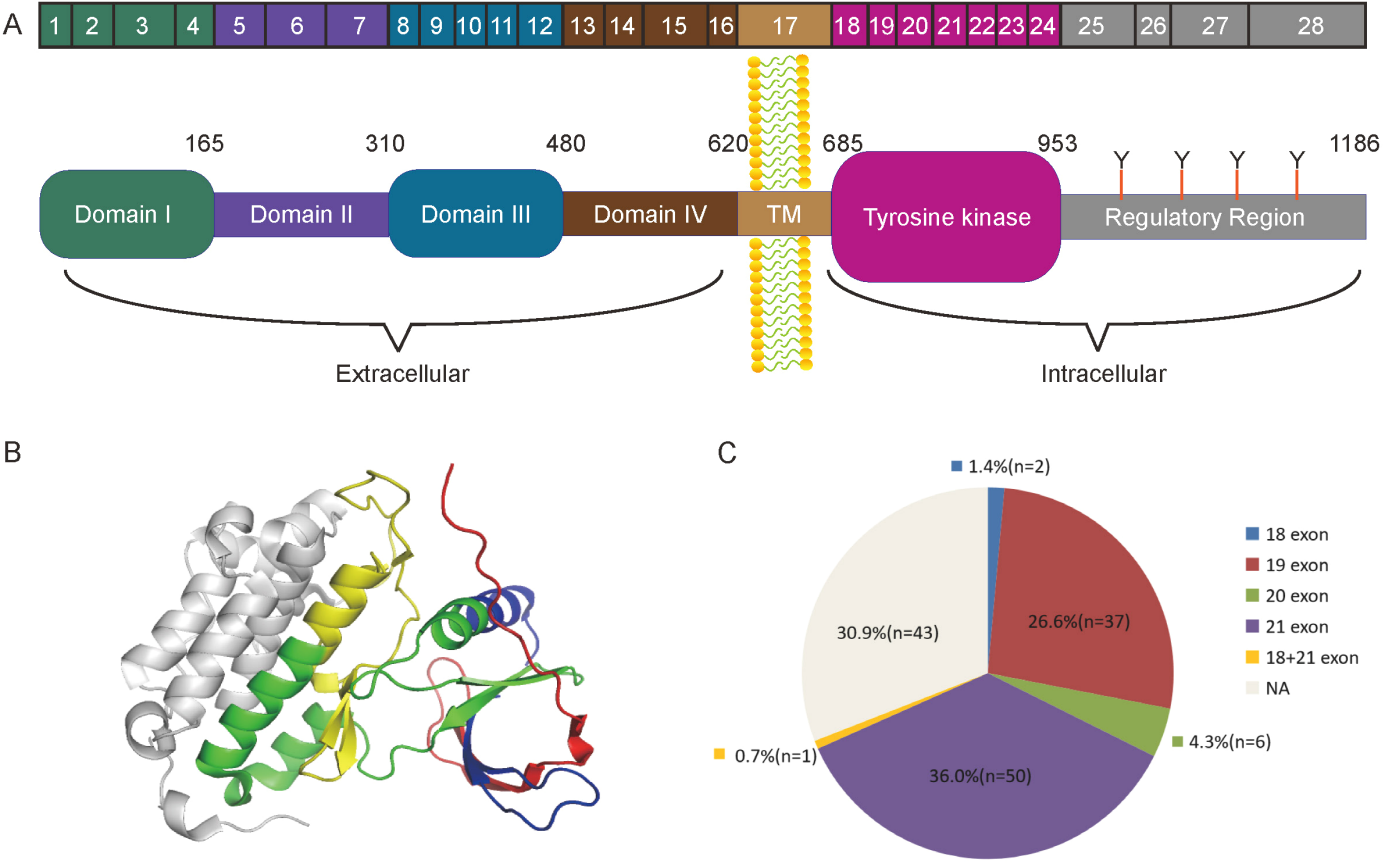
The structure of EGFR and the mutation sites related to lung adenocarcinoma. **(A)** Complete Structure diagram of the EGFR gene. The diagram showed different domains and exons of EGFR. **(B)** The structure of the EGFR tyrosine kinase domain. Exons18-21 were marked sequentially as red, blue, green and yellow. **(C)** The composition diagram of EGFR mutation in the cohort.

The common detected mutations of EGFR in lung cancer range from exon 18 to exon 21 (**Figures 4A, B**). We detected these EGFR mutations in the cohort, and found that EGFR mutations occurred in 96 (69.1%) cases. In these mutated cases, the most common EGFR mutation was a missense mutation (L858R) in exon 21 (47/96, 49.0%) and the second most common was an in-frame deletion in exon 19 (37/96, 38.5%) (**Figure 4C**). The detailed information of EGFR mutations in the cohort was shown in **Tables 1**, **2**. EGFR mutations were more frequently occurred in never smokers (73.2% vs. former or current smokers 51.9%; P=0.0311) and patients with tumor diameter larger than 1.5cm (78.5% vs. tumor diameter less than 1.5cm 60.8%; P = 0.0247). In this study, the EGFR mutations had no significant correlation with gender, age, tumor location, hypertension, diabetes mellitus, family history and tumor counts.

**Table 2:** The detailed information of EGFR mutations in the cohort.

Gene	Exon	Amino acid change	N (%)
*EGFR*	18	G719X	2 (2.08%)
	19	Amino acid deletion	37 (38.54%)
	20	V769_D770insASV	4 (4.17%)
		S768I	2 (2.08%)
	21	L858R	47 (48.96%)
		L861Q	3 (3.13%)
	18 + 21	G719X 、L858R	1 (1.04%)

### Correlation between EGFR mutations and pathological features

**Figure 5:**
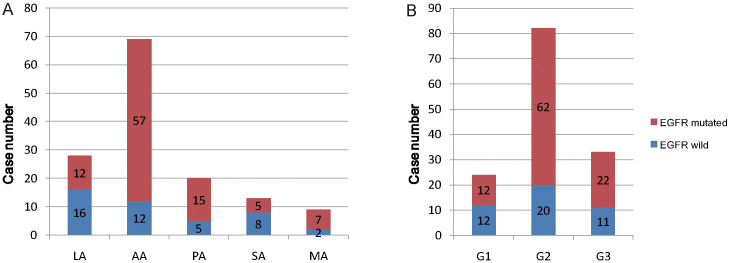
The relationship diagram of EGFR mutation and pathological features. **(A)** EGFR mutation proportion in five histological subtypes of lung adenocarcinoma. **(B)** EGFR mutation proportion in three histological grades of lung adenocarcinoma. G1, G2, G3 is short for histological grade 1, grade 2 and grade 3, corresponding to well differentiation, moderately differentiation and poorly differentiation.

Five histological subtypes of lung adenocarcinoma included lepidic subtype, acinar subtype, papillary subtype, solid subtype and micropapillary subtype. In this research, the EGFR mutation rate was significantly related to the histological subtypes of lung adenocarcinoma (P=0.0003) (**Table 3**). Furthermore, the acinar subtype adenocarcinoma had a higher EGFR mutation rate than solid subtype adenocarcinoma (P=0.0007) and lepidic subtype adenocarcinoma (P<0.0001) (**Table 3**, **Figure 5A**). Also, the papillary subtype adenocarcinoma had a higher EGFR mutation rate than solid (P=0.0358) and lepidic (P=0.0269) subtype adenocarcinoma (**Table 3**, **Figure 5A**). Although in this study, the EGFR mutation rate was almost unrelated to the differentiation grade (P=0.0546), the incidence rate of EGFR mutation in patients with moderately differentiated lung adenocarcinoma was higher than patients with well differentiated adenocarcinoma (P =0.0162) (**Table 3**, **Figure 5B**).

**Table 3:** The association between EGFR mutations and the histological characteristics.

	N	Mutant EGFR	Wild type EGFR	P value
**Histological Subtypes**				***0.0003***
LA	28	12	16	
AA	69	57	12	
PA	20	15	5	
SA	13	5	8	
MA	9	7	2	
**Differentiation**				0.0546
Well-differentiated (G1)	24	12	12	
moderately differentiated (G2)	82	62	20	
Poorly differentiated (G3)	33	22	11	
**Lymphocytes infiltration**				0.718
Yes	94	64	30	
No	45	32	13	
**Extracellular mucus**				***0.0013***
Yes	17	6	11	
No	122	90	32	
**Fibrosis**				0.9618
Yes	78	54	24	
No	61	42	19	
**Mucous epithelium**				0.5482
Yes	22	14	8	
No	117	82	35	
**Necrosis**				0.6848
Yes	11	7	4	
No	128	89	39	
**Abscess**				0.1759
Yes	3	1	2	
No	136	95	41	
**Hobnail cell**				0.2842
Yes	43	27	16	
No	96	69	27	
**rhabdomyoid differentiation**				***0.0237***
Yes	25	22	3	
No	114	74	40	
**STAS**				0.4448
Yes	27	17	10	
No	112	79	33	
**Mitosis**				0.3454
<1/10HPF	89	59	30	
≥1/10HPF	50	37	13	
**Nucleolus**				0.6022
Yes	33	24	9	
No	106	72	34	
**High grade nuclear**				0.2107
Yes	39	30	9	
No	100	66	34	
**Nuclear inclusion**				0.1184
Yes	15	13	2	
No	124	83	41	
**Pleural invasion**				0.4698
Yes	38	28	10	
No	101	68	33	
**Vessel invasion**				0.1758
Yes	10	5	5	
No	129	91	38	
**Ki67**				0.8095
<10%	42	29	13	
≥10%	48	32	16	

The bold and italic values mean statistically significant.

**Figure 6:**
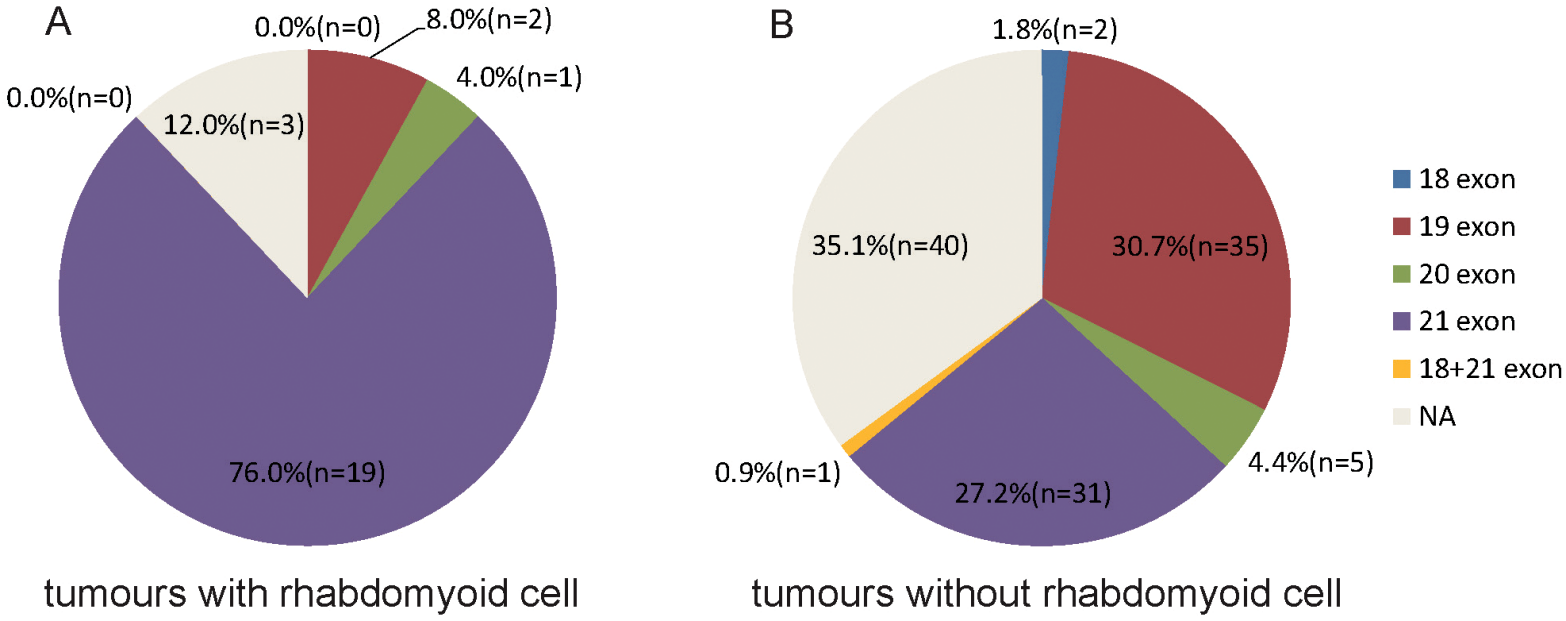
Percentage of EGFR mutation location of lung adenocarcinoma with **(A)** and without **(B)** rhabdomyoid differentiation.

Significantly, EGFR mutation occurred more frequently in tumors with rhabdomyoid differentiation (88.0% vs. tumors without rhabdomyoid differentiation, 64.9%; P = 0.0237) and tumors without extracellular mucus (73.8% vs. tumors with extracellular mucus, 35.3%; P = 0.0013) (**Table 3**). Furthermore, the exon 21 mutation occurred more frequently in tumors with rhabdomyoid differentiation (86.4% vs. tumors without rhabdomyoid differentiation, 41.9%; P =0.0077) (**Table 4**, **Figure 6**). However, the EGFR mutation rate had no relationship with the proliferation index (Ki67 expression ratio). The detailed correlation information of EGFR mutations and histological features were shown in **Tables 3**, **4**.

**Table 4:** The correlation between EGFR mutation and the Rhabdomyoid feature.

	N	With rhabdomyoid differentiation	Without rhabdomyoid differentiation	P value
**Mutant EGFR**				***0.0077***
EGFR18 exon	2	0	2	
EGFR19 exon	37	2	35	
EGFR20 exon	6	1	5	
EGFR21 exon	50	19	31	
EGFR18 + 21 exon	1	0	1	
**Wild type EGFR**	43	3	40	

The bold and italic values mean statistically significant.

## Discussion

EGFR mutation is known to be the most common mutation type of lung adenocarcinoma, which is associated with specific clinical features. In this study, all the patients were of East Asian ethnicity. The EGFR mutation rate was 69.1% (96/139), much higher than that of Caucasian patients (33–36). Previously research indicated that EGFR mutations occurred more frequently in women, never smokers and East Asian ethnicity (8–15, 37, 38). Consistent with this, EGFR mutations occurred more frequently in never smokers (P=0.0311) in this research. But inconsistent with previous researches, there was no significant difference in EGFR mutation incidence between women and men (P=0.1690). Previous studies showed that EGFR mutation preferentially occurred in patients with maximal tumor size less than 3 cm (14). In our research, the median tumor diameter was 1.5cm, which was selected as the cut-off value. The EGFR mutation occurred more frequently in tumors with a diameter larger than 1.5cm(P=0.0247). There was no more statistical correlation between EGFR mutation rate and other clinical features (**Table 1**).

According to the IASLC/ATS/ERS classification criteria, the non-mucinous lung adenocarcinoma could be divided into five histological subtypes and three differentiation grades. The classification criteria were mainly based on tumor morphology, lacking the study of molecular changes. Nowadays, the correlation between molecular changes and morphology characteristics of lung adenocarcinoma has attracted the research interests greatly, especially the EGFR mutations. Recent research showed that the lung adenocarcinoma with solid subtype had a lower EGFR mutation rate (39–42). The other research indicated that EGFR mutations occurred most frequently in acinar lung adenocarcinoma and least frequently in solid lung adenocarcinoma (43). Consistently, in our study, lung adenocarcinoma with acinar and papillary subtype had a higher EGFR mutation rate than solid and lepidic subtype. In addition, we found that the EGFR mutation occurred more frequently in moderately differentiated lung adenocarcinoma than well differentiated adenocarcinoma.

The lung adenocarcinoma has heterogenous histological characteristics, such as cell atypia, nuclear characteristics, cytoplasmic morphology, interstitial abnormality, mitosis rate, and so on. In this research, we also explored the correlation between EGFR mutation and these morphological features.

Interestingly, we found that EGFR mutation occurred more frequently in adenocarcinoma with rhabdomyoid differentiation. Besides, EGFR mutation of the adenocarcinoma with rhabdomyoid differentiation mainly occurred at exon 21. Our research indicated that EGFR mutations at exon 21 may result in rhabdomyoid differentiation, which connected the genetic changes with the morphological characteristics of lung adenocarcinoma. Lung adenocarcinomas with rhabdoid differentiation are morphologically similar to rhabdoid cells, but are essentially lung adenocarcinomas. They still express lung adenocarcinoma-specific immunohistochemical markers, such as TTF-1 and NapsinA, but do not express myogenic immunohistochemical markers, such as MyoD-1 or Desmin.Several studies have shown that meningiomas and renal cell carcinomas with rhabdoid differentiation have more aggressive biological behaviors and poor prognosis (44, 45).However, there is no report on lung adenocarcinoma with rhabdoid differentiation, neither on the relationship between EGFR mutation and lung adenocarcinoma with rhabdoid differentiation.The mechanism of rhabdoid differentiation is unknown and needs further study.

We also found that the non-mucous adenocarcinomas with extracellular mucus had a lower EGFR mutation rate. The pathogenesis of lung adenocarcinoma and invasive mucinous adenocarcinoma (IMA) was totally different. The IMA had a much lower EGFR rate than typical lung adenocarcinoma (46, 47). The lung adenocarcinoma with extracellular mucus may be a complex of typical lung adenocarcinoma and IMA. Therefore, the genetic characteristics also may be affected.

## Conclusions

In summary, our research revealed that the EGFR mutation state was associated with smoking history, histological subtypes, tumor size, and some rare histological features, such as rhabdomyoid differentiation and extracellular mucus. In-depth study of morphological characteristics is conducive to early prediction of genetic changes, which is meaningful to postoperative chemotherapy.

## Data Availability

The original contributions presented in the study are included in the article/supplementary material. Further inquiries can be directed to the corresponding author/s.

## References

[1] Miller KD Nogueira L Devasia T Mariotto AB Yabroff KR Jemal A. Cancer treatment and survivorship statistics, 2022. CA Cancer J Clin. (2022) 72:409–36. doi: 10.3322/caac.21731 35736631

[2] Siegel RL Giaquinto AN Jemal A. Cancer statistics, 2024. CA Cancer J Clin. (2024) 74:12–49. doi: 10.3322/caac.21820 38230766

[3] Huang Z Chen X Wang Y Yuan J Li J Hang W. SLC7A11 inhibits ferroptosis and downregulates PD-L1 levels in lung adenocarcinoma. Front Immunol. (2024) 15:1372215. doi: 10.3389/fimmu.2024.1372215 38655266 PMC11035808

[4] Travis WD Brambilla E Noguchi M Nicholson AG Geisinger KR Yatabe Y. International association for the study of lung cancer/american thoracic society/european respiratory society international multidisciplinary classification of lung adenocarcinoma. J Thorac Oncol. (2011) 6:244–85. doi: 10.1097/JTO.0b013e318206a221 PMC4513953 21252716

[5] Travis BE Nicholson WD Borczuk AG Noguchi AC Galateau-Sallé M Cooper WA. WHO classification of tumours of the lung, pleura, thymus and heart. 5th ed. 69372 Lyon Cedex 08, France: International Agency for Research on Cancer Press (2021).

[6] Parker MI Nikonova AS Sun D Golemis EA. Proliferative signaling by ERBB proteins and RAF/MEK/ERK effectors in polycystic kidney disease. Cell Signal. (2020) 67:109497. doi: 10.1016/j.cellsig.2019.109497 31830556 PMC6957738

[7] Boch T Kohler J Janning M Loges S. Targeting the EGF receptor family in non-small cell lung cancer-increased complexity and future perspectives. Cancer Biol Med. (2022) 19:1543–64. doi: 10.20892/j.issn.2095-3941.2022.0540 PMC9724226 36476337

[8] Hill W Lim EL Weeden CE Lee C Augustine M Chen K. Lung adenocarcinoma promotion by air pollutants. Nature. (2023) 616:159–67. doi: 10.1038/s41586-023-05874-3 PMC7614604 37020004

[9] Park SJ Ju S Goh SH Yoon BH Park JL Kim JH. Proteogenomic characterization reveals estrogen signaling as a target for never-smoker lung adenocarcinoma patients without EGFR or ALK alterations. Cancer Res. (2024) 84:1491–503. doi: 10.1158/0008-5472.CAN-23-1551 38607364

[10] He D Wang D Lu P Yang N Xue Z Zhu X. Single-cell RNA sequencing reveals heterogeneous tumor and immune cell populations in early-stage lung adenocarcinomas harboring EGFR mutations. Oncogene. (2021) 40:355–68. doi: 10.1038/s41388-020-01528-0 PMC7808940 33144684

[11] Komurcuoglu B Karakurt G Kaya OO Diniz G Kirbiyik O Evkan A. Investigation of EGFR and ALK mutation frequency and treatment results in advanced non-small cell lung cancer. J Cancer Res Ther. (2023) 19:S183–90. doi: 10.4103/jcrt.JCRT_1766_20 37147996

[12] Li D Ding L Ran W Huang Y Li G Wang C. Status of 10 targeted genes of non-small cell lung cancer in eastern China: A study of 884 patients based on NGS in a single institution. Thorac Cancer. (2020) 11:2580–9. doi: 10.1111/1759-7714.13577 PMC7471050 32729257

[13] Wang T Zhang Y Liu B Hu M Zhou N Zhi X. Associations between epidermal growth factor receptor mutations and histological subtypes of lung adenocarcinoma according to the IASLC/ATS/ERS classification in Chinese patients. Thorac Cancer. (2017) 8:600–5. doi: 10.1111/tca.2017.8.issue-6 PMC5668489 28940943

[14] Wang H Zhang W Wang K Li X. Correlation between EML4-ALK, EGFR and clinicopathological features based on IASLC/ATS/ERS classification of lung adenocarcinoma. Med (Baltimore). (2018) 97:e11116. doi: 10.1097/MD.0000000000011116 PMC6039605 29952952

[15] Wen L Wang S Xu W Xu X Li M Zhang Y. Value of serum tumor markers for predicting EGFR mutations in non-small cell lung cancer patients. Ann Diagn Pathol. (2020) 49:151633. doi: 10.1016/j.anndiagpath.2020.151633 32977235

[16] Pretelli G Spagnolo CC Ciappina G Santarpia M Pasello G. Overview on therapeutic options in uncommon EGFR mutant non-small cell lung cancer (NSCLC): new lights for an unmet medical need. Int J Mol Sci. (2023) 24. doi: 10.3390/ijms24108878 PMC10218597 37240224

[17] Chung C. Tyrosine kinase inhibitors for epidermal growth factor receptor gene mutation-positive non-small cell lung cancers: an update for recent advances in therapeutics. J Oncol Pharm Pract. (2016) 22:461–76. doi: 10.1177/1078155215577810 25855240

[18] Saleem TH Elkhayat H Farouk A Gabra FA Omar EA Kamel AA. Evaluation of the role of EGFR exon 19 747-750 deletion mutation and plasma amino acid profile in the development of lung cancer. Mol Biol Rep. (2024) 51:1039. doi: 10.1007/s11033-024-09941-4 39367097

[19] Liu Q Yu S Zhao W Qin S Chu Q Wu K. EGFR-TKIs resistance via EGFR-independent signaling pathways. Mol Cancer. (2018) 17:53. doi: 10.1186/s12943-018-0793-1 29455669 PMC5817859

[20] Singh M Jadhav HR. Targeting non-small cell lung cancer with small-molecule EGFR tyrosine kinase inhibitors. Drug Discovery Today. (2018) 23:745–53. doi: 10.1016/j.drudis.2017.10.004 29031620

[21] Rybarczyk-Kasiuchnicz A Ramlau R Stencel K. Treatment of brain metastases of non-small cell lung carcinoma. Int J Mol Sci. (2021) 22. doi: 10.3390/ijms22020593 PMC7826874 33435596

[22] Wang YT Yang PC Zhang JY Sun JF. Synthetic routes and clinical application of representative small-molecule EGFR inhibitors for cancer therapy. Molecules. (2024) 29. doi: 10.3390/molecules29071448 PMC11012680 38611728

[23] Lin Z Li J Zhang J Feng W Lu J Ma X. Metabolic reprogramming driven by IGF2BP3 promotes acquired resistance to EGFR inhibitors in non-small cell lung cancer. Cancer Res. (2023) 83:2187–207. doi: 10.1158/0008-5472.CAN-22-3059 37061993

[24] Huang L Huang H Zhou XP Liu JF Li CR Fang M. Osimertinib or EGFR-TKIs/chemotherapy in patients with EGFR-mutated advanced nonsmall cell lung cancer: A meta-analysis. Med (Baltimore). (2019) 98:e17705. doi: 10.1097/MD.0000000000017705 PMC6824777 31651902

[25] Chen Y Wen S Wu Y Shi L Xu X Shen B. Efficacy and safety of first-generation epidermal growth factor receptor (EGFR) tyrosine kinase inhibitors (TKIs) combined with chemotherapy or antiangiogenic therapy as first-line treatment in patients with EGFR-mutant non-small cell lung cancer: A systematic review and meta-analysis. Crit Rev Oncol Hematol. (2021) 163:103393. doi: 10.1016/j.critrevonc.2021.103393 34119658

[26] Hu L Xie X. Parathyroid carcinoma with sarcomatoid differentiation: a case report and literature review. Diagn Pathol. (2020) 15:142. doi: 10.1186/s13000-020-01060-5 33317559 PMC7737283

[27] Narayan P Kostrzewa CE Zhang Z O'Brien DAR Mueller BA Cuaron JJ. Metaplastic carcinoma of the breast: matched cohort analysis of recurrence and survival. Breast Cancer Res Treat. (2023) 199:355–61. doi: 10.1007/s10549-023-06923-1 PMC12529652 36976395

[28] Wang XJ Wang CY Xi YF Bu P Wang P. Ovarian mucinous tumor with mural nodules of anaplastic carcinoma: Three case reports. World J Clin cases. (2022) 10:7459–66. doi: 10.12998/wjcc.v10.i21.7459 PMC9353926 36158006

[29] Ma H Shi S Zhang Z Liu H. Primary signet−ring cell melanoma of the anorectum: A case report. Oncol Lett. (2023) 25:220. doi: 10.3892/ol.2023.13806 37153063 PMC10157357

[30] Wang AX Tian T Liu LB Yang F He HY Zhou LQ. TFEB rearranged renal cell carcinoma: pathological and molecular characterization of 10 cases, with novel clinical implications: A single center 10-year experience. Biomedicines. (2023) 11. doi: 10.3390/biomedicines11020245 PMC9952947 36830782

[31] Zhao JP Cui CX Wang JC Su HW Duan CF Liu XJ. Multimodal MR features of 8 cases of epithelioid glioblastoma. BioMed Res Int 2020. (2020), 9586806. doi: 10.1155/2020/9586806 PMC7586153 33123592

[32] Wang H Zhou C Su B Teng G Zheng Y Zhou X. MCM7 expression is correlated with histological subtypes of lung adenocarcinoma and predictive of poor prognosis. Int J Clin Exp Pathol. (2017) 10:11747–53. PMC6966067 31966536

[33] Mansuet-Lupo A Bobbio A Blons H Becht E Ouakrim H Didelot A. The new histologic classification of lung primary adenocarcinoma subtypes is a reliable prognostic marker and identifies tumors with different mutation status: the experience of a French cohort. Chest. (2014) 146:633–43. doi: 10.1378/chest.13-2499 24676429

[34] Warth A Muley T Meister M Stenzinger A Thomas M Schirmacher P. The novel histologic International Association for the Study of Lung Cancer/American Thoracic Society/European Respiratory Society classification system of lung adenocarcinoma is a stage-independent predictor of survival. J Clin Oncol. (2012) 30:1438–46. doi: 10.1200/JCO.2011.37.2185 22393100

[35] Kobayashi Y Mitsudomi T. Not all epidermal growth factor receptor mutations in lung cancer are created equal: Perspectives for individualized treatment strategy. Cancer Sci. (2016) 107:1179–86. doi: 10.1111/cas.2016.107.issue-9 PMC5021039 27323238

[36] Lamb YN. Osimertinib: A review in previously untreated, EGFR mutation-positive, advanced NSCLC. Target Oncol. (2021) 16:687–95. doi: 10.1007/s11523-021-00839-w PMC8536603 34564820

[37] Yang ZM Ding XP Pen L Mei L Liu T. Analysis of CEA expression and EGFR mutation status in non-small cell lung cancers. Asian Pac J Cancer Prev. (2014) 15:3451–5. doi: 10.7314/APJCP.2014.15.8.3451 24870738

[38] Zhang Y Sun Y Pan Y Li C Shen L Li Y. Frequency of driver mutations in lung adenocarcinoma from female never-smokers varies with histologic subtypes and age at diagnosis. Clin Cancer Res. (2012) 18:1947–53. doi: 10.1158/1078-0432.CCR-11-2511 PMC3319848 22317764

[39] Song Z Zhu H Guo Z Wu W Sun W Zhang Y. Correlation of EGFR mutation and predominant histologic subtype according to the new lung adenocarcinoma classification in Chinese patients. Med Oncol. (2013) 30:645. doi: 10.1007/s12032-013-0645-1 23797772

[40] Boukansa S Benbrahim Z Gamrani S Bardai S Bouguenouch L Mazti A. Correlation of epidermal growth factor receptor mutation with major histologic subtype of lung adenocarcinoma according to IASLC/ATS/ERS classification. Cancer Control. (2022) 29:10732748221084930. doi: 10.1177/10732748221084930 35348028 PMC8969502

[41] Clay TD Russell PA Do H Sundararajan V Conron M Wright GM. Associations between the IASLC/ATS/ERS lung adenocarcinoma classification and EGFR and KRAS mutations. Pathology. (2016) 48:17–24. doi: 10.1016/j.pathol.2015.11.002 27020204

[42] Deng H Liu J Duan X Liu Y. The relationship between EGFR mutation status and clinic-pathologic features in pulmonary adenocarcinoma. Pathol Res Pract. (2018) 214:450–4. doi: 10.1016/j.prp.2017.09.008 29496311

[43] Nakamura H Saji H Shinmyo T Tagaya R Kurimoto N Koizumi H. Association of IASLC/ATS/ERS histologic subtypes of lung adenocarcinoma with epidermal growth factor receptor mutations in 320 resected cases. Clin Lung Cancer. (2015) 16:209–15. doi: 10.1016/j.cllc.2014.10.004 25467929

[44] Garrido Ruiz PA Gonzalez-Tablas M Pena Pasco A Huerta Zelaya MV Ortiz J Otero A. Clinical, histopathologic and genetic features of rhabdoid meningiomas. Int J Mol Sci. (2023) 24. doi: 10.3390/ijms24021116 PMC9865044 36674634

[45] Yang B Xia H Xu C Lu M Zhang S Wang G. Impact of sarcomatoid differentiation and rhabdoid differentiation on prognosis for renal cell carcinoma with vena caval tumour thrombus treated surgically. BMC Urol. (2020) 20:14. doi: 10.1186/s12894-020-0584-z 32070319 PMC7029456

[46] Kadota K Yeh YC D'Angelo SP Moreira AL Kuk D Sima CS. Associations between mutations and histologic patterns of mucin in lung adenocarcinoma: invasive mucinous pattern and extracellular mucin are associated with KRAS mutation. Am J Surg Pathol. (2014) 38:1118–27. doi: 10.1097/PAS.0000000000000246 PMC4666292 25029118

[47] Cai L Wang J Yan J Zeng J Zhu L Liang J. Genomic profiling and prognostic value analysis of genetic alterations in chinese resected lung cancer with invasive mucinous adenocarcinoma. Front Oncol. (2020) 10:603671. doi: 10.3389/fonc.2020.603671 33505917 PMC7829865

